# Procedural Treatments for Knee Osteoarthritis: A Review of Current Injectable Therapies

**DOI:** 10.1155/2020/3873098

**Published:** 2020-02-18

**Authors:** Lisa M. Billesberger, Kyle M. Fisher, Yawar J. Qadri, Richard L. Boortz-Marx

**Affiliations:** ^1^Private Practice, London, ON, Canada; ^2^Duke Anesthesiology, Duke University School of Medicine, Durham, USA; ^3^Duke Pain Medicine, Durham, USA

## Abstract

Knee osteoarthritis is a common painful degenerative condition affecting the aging Canadian population. In addition to pain and disability, osteoarthritis is associated with depression, comorbid conditions such as diabetes, and increased caregiver burden. It is predicted to cost the Canadian healthcare system $7.6 billion dollars by 2031. Despite its high cost and prevalence, controversy persists in the medical community regarding optimal therapies to treat knee osteoarthritis. A variety of medications like nonsteroidal anti-inflammatories and opioids can cause severe side effects with limited benefits. Total knee arthroplasty, although a definitive management, comes with risk such as postoperative infections, revisions, and chronic pain. Newer injectable therapies are gaining attention as alternatives to medications because of a safer side effect profile and are much less invasive than a joint replacement. Platelet-rich plasma is beginning to replace the more common injectable therapies of intra-articular corticosteroids and hyaluronic acid, but larger trials are needed to confirm this effect. Small studies have examined prolotherapy and stem cell therapy and demonstrate some benefits. Trials involving genicular nerve block procedures have been successful. As treatments evolve, injectable therapies may offer a safe and effective pathway for patients suffering from knee osteoarthritis.

## 1. Introduction

Osteoarthritis (OA) in Canada has a marked impact on patient quality of life and comorbid conditions as well as a dramatic economic cost. Sharif et al. estimated that, by 2031, the productivity costs of work loss related to OA would total nearly $17.5 billion dollars [1]. As a chronic disease often associated with aging, the burden of OA will continue to increase as the Canadian population ages. In 2010, 15% of the population was 65 years old or older, and by 2031, 25% of the population is estimated to be 65 years old or older [2]. Thus, offering directed treatments that are safe, cost effective, and beneficial to the patient will be of paramount importance. Topical analgesia, as well as oral analgesics such as acetaminophen, nonsteroidal anti-inflammatories, duloxetine, and opioids have associated side effects [3] and dubious efficacy, with a well-powered meta-analysis demonstrating a considerable uncertainty regarding the efficacy of oral medications for knee-OA (K-OA) as compared to placebos [4]. Opioid use, in particular, is becoming increasingly ostracized as we begin to appreciate its negative impact on both the individual patient as well as society in general. Additionally, the effect of opioid medications compared to nonopioid medications showed negligible effect on pain after 12 months of treatment [5]. Thus, for the savvy clinician, we will review nonsurgical treatments for K-OA such as injections, nerve blocks, and the so-called “regenerative” medicines: corticosteroid (CS) injections, hyaluronic acid (HA) injections, platelet-rich plasma (PRP) injections, prolotherapy, genicular nerve blocks, and stem cell therapy. These therapies are often viewed as the last stop prior to operative intervention and are of particular importance [6]. Current society recommendations for OA treatment modalities, where available, are summarized [7–9] (Table 1).

## 2. The Scope of the Problem

The Canadian healthcare system will only be sustainable if we are able to offer quality care when it counts or, as Bronskill et al. eloquently state, “the right care, at the right time, and in the right place” [2]. As the Canadian population ages and becomes ever more obese and inactive, the rate of OA is also rising and is projected by the Population Health Microsimulation Model of Osteoarthritis (POHEM-OA) to increase from 13.8% to 18.6% of the total population between 2010 and 2031 [10]. Factoring in the cost of physician visits, hospitalization, rehabilitation, medications, and side effects of medications, the total direct cost of OA to the patient and the healthcare system is expected to increase by 160% from $2.9 billion to $7.6 billion. However, there are also numerous indirect costs associated with OA, not just to the patient, but also to caregivers [11]. When including the associated costs of lost productivity from OA, the economic burden of OA becomes even more substantial. OA significantly impacts premature retirement, long-term sick leave or disability, and reduces employment [1].

**Figure 1:**
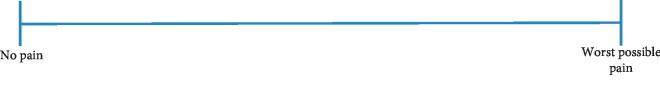
The Visual Analog Scale (VAS), a common measurement tool for pain research. The VAS is a 100 mm long line. A patient will place a single vertical mark on the scale to indicate the intensity of their pain. The mark is then measured and recorded by the caregiver.

The impact of OA on the Canadian population is not limited solely to economic burden. Depression is commonly associated with OA, and patients who are depressed have worse outcomes in both operative and nonoperative treatment of OA [12–14]. A 2015 review of Ontario patients by Gandhi et al. found that patient reporting depression increased in frequency by 19% and correlated with each successive higher painful joint score from 0–10 [12]. In addition to depression, patients with OA also report a reduction in ability to move, sexual activity, and vitality compared to population-matched controls [15, 16]. Interestingly, a 2018 population-based cohort study including 362 Ontario patients demonstrated that, even after controlling for confounders, the presence and burden of knee and hip OA was a powerful independent predictor of type 2 diabetes [17]. Numerous studies have examined the impact of wait time for joint replacement on health-related quality of life (HRQoL) using two commonly accepted HRQoL measurement tools—the Western Ontario and McMaster Osteoarthritis Index (WOMAC) and the Visual Analog Score (VAS) (Table 2 and Figure 1). In sum, longer wait times to joint replacement seem to correlate with decreasing HRQoL [6, 18–20]. Given that OA, and specifically K-OA, have such a negative impact, it is not surprising that total knee arthroplasty (TKA) has become one of the most common surgical procedures in Canada with 67,000 total knee replacements performed from 2016 to 2017 [21].

TKAs cost more than nonsurgical treatment alone. However, over a patient's lifetime, they reduce indirect costs, and one study estimated that a TKA can net a cost savings of $18930 per patient [22]. In the USA, TKAs in 2009 provided an estimated $12 billion of estimated societal savings [23]. A 2015 Danish study published in the New England Journal of Medicine found that, when compared to lifestyle interventions alone, TKA followed by lifestyle interventions improved pain, function, and quality of life. The cost of this improvement, however, was steep, and patients receiving TKA had markedly increased risk of adverse events. Thus, TKAs seem to be beneficial only when the potential benefit outweighs the risk and thus varies from patient to patient [24]. Some studies have estimated that up to 25% of patients receiving TKAs were inappropriately provided with this treatment [15, 25–28]. Furthermore, up to one third of patients continue to experience chronic knee pain after TKA [29]. Arthroscopy is another common treatment for treating K-OA; however, it remains controversial with some authors showing it to only provide transient, inconsequential benefit with an unacceptably high risk of adverse events [30]. Interestingly, other authors have found arthroscopy to temporize TKA by a mean of 6.8 years and over ten years in 40% of patients [31].

## 3. Knee Anatomy

The knee comprises bony structures, ligaments, cartilage, and synovial membrane. Synovial fluid, produced by the synovial membrane, lubricates the joint and provides the avascular cartilage with nutrients. Treatment of OA can be hampered by the avascular and aneural nature of the knee joint cartilage [29]. K-OA is typically classified as either primary/idiopathic or secondary due to trauma or misalignment. In contrast to the dated view that OA is purely a degenerative process, the current multimodal understanding of OA includes trauma, mechanical forces, biochemical cartilage degradation, inflammation, and metabolic derangements [32]. Unlike the inflammation seen in inflammatory arthritis, inflammation in OA is chronic and low-grade [32]. In early OA, the damage to the cartilage itself is not able to produce an inflammatory response which causes pain. However, as OA progresses, the bony and ligamentous structures are impacted, undergoing bony remodeling, osteophyte formation, and ligamentous laxity [32, 33]. Additionally, a synovial effusion forms. It is not certain whether the effusion is a result of OA or if the effusion leads to OA [34], although K-OA can sometimes be differentiated in to either a “wet” or “dry” OA, referring to having an effusion or no effusion, respectively [35]. We know that when present this effusion is rich in inflammatory mediators, including C-reactive protein (CRP), prostaglandins (PGE2), cytokines (TNF, IL-1B, IL-6, IL1-5, IL-17, IL-18, and IL-21), leukotrienes (LKB4), growth factors (TGF-B and VEGF), complement proteins, and nitric oxide [35, 36]. Together, this rich inflammatory milieu results in breakdown of the cartilage. Innate immune cells recognize certain damage-associated molecular patterns released by the breakdown of the extracellular matrix through pattern recognition receptors, leading to further tissue destruction and remodeling. As we learn more about the pathophysiology of OA, we can continue to direct our therapies to stopping OA at the source rather than reducing the pain with analgesics.

## 4. Corticosteroid Injections

The leading societies on osteoarthritis management continue to debate the usefulness of intra-articular (I-CS) injections, and each come to a different conclusion (Table 1). Nonetheless, I-CS injections have been widely used for decades to treat K-OA, and specialists and general practitioners across the country perform this procedure daily. Steroids act as local anti-inflammatory medications and are thought to counteract the inflammatory processes in K-OA by altering T- and B-cell immune function [37, 38]. Synthetic corticosteroids exhibit more anti-inflammatory effect than their native counterparts, and methylprednisolone acetate and triamcinolone are the most commonly used I-CS for K-OA [39].

Many clinicians believe that the benefits of I-CS continue to outweigh the risk of complications. Yet, the evidence in the literature makes the use of I-CS injections controversial. A recent Cochrane review regarding I-CS for K-OA assessed 27 low-quality and heterogeneous RCT's that included 1767 patients [37]. At one month post-injection, pain scores in the I-CS group compared to placebo improved by only 1.0 cm on the VAS score, corresponding to a number needed to treat of 8 (95% CI 6–13). Furthermore, at 13 weeks, steroid injections provided an even smaller benefit in pain control, and the effect was absent by 26 weeks. The Cochrane review also used the WOMAC to assess function after I-CS injection. Overall, a moderate improvement at 1-2 weeks was detected, but the effect declined by 4–6 weeks and was all gone by 13 weeks. The authors concluded that the poor quality heterogeneous studies make it difficult to determine if there is an early benefit in pain relief or function with I-CS compared to placebo [37]. Thus, despite speculation of early pain relief, the effect is not long lasting. Adverse events in I-CS injections do occur. A post-injection flare-up of pain can occur in 2–25% of patients and last for a few days [40]. Also, there may also be progression of the K-OA, with a 2017 RCT determining that intra-articular injection with triamcinolone resulted in greater cartilage volume loss when compared to intra-articular saline injection at two years [41]. Systemic side effects of corticosteroids include elevated blood pressure, hyperglycemia, and alterations in mood and energy [29]. Triamcinolone, which is less water soluble than methylprednisolone, may be a better alternative for those with diabetes and in whom the risk of post-injection hyperglycemic spikes should be minimized [40]. Skin depigmentation, fat necrosis, and cutaneous atrophy are also possible after I-CS, but are observed much less commonly [40, 42]. Despite a theoretical concern for infection, many older studies done over 40 years ago have debunked this risk. [37, 43–47]. Interestingly, a newer study in 2017 suggested that I-CS before TKA might increase the risk of postoperative infection [39]. With the growing evidence of I-CS having only a mild and transient effect on pain relief, one must question the sustainability of this therapy in modern day evidence-based medicine.

## 5. Hyaluronic Acid Injections

HA, the main ingredient of synovial fluid, is a glycosaminoglycan that is produced by synoviocytes, chondrocytes, and fibroblasts [35]. Healthy knees contain between 2.5 and 4 mg/mL of HA, while osteoarthritic knees are up to 50% deficient in HA secondary to decreased production, degradation, and increased clearance [48, 49]. The mechanism of action for injections of intra-articular HA (I-HA) is not well understood, but is postulated to reduce friction and improve elasticity and the shock absorption of the knee joint [50]. Interestingly, it might also attenuate phagocytosis, thus reducing tissue destruction, as well as inhibiting nociceptors [35, 49]. Some researchers have suggested that I-HA can be used preferentially to treat those with “dry” OA [50]. Despite being approved by the FDA in 1997, the use of I-HA for the treatment of K-OA remains controversial because of conflicting data regarding its efficacy reached by various meta-analyses and reviews. This is reflected in the varying recommendations reached by each society for osteoarthritis management (Table 1).

A 2015 systemic review of 14 meta-analyses found that the highest level of evidence demonstrated “viability” for I-HA in K-OA [51]. Unfortunately, the control was not the same for each meta-analysis included, therefore reducing the quality of the evidence. Of the studies included that compared I-HA to intra-articular placebo injection, the results were lackluster. Some demonstrated improvement with I-HA, while others did not [51]. Three studies examined I-HA compared to oral NSAIDs and found that there was no significant difference in outcome [51]. Importantly, however, I-HA had fewer negative side effects compared to oral NSAIDs. When compared to I-CS, which provided the most pain relief during the first 4 weeks post-injection, I-HA demonstrated better outcomes at 5 and 13 weeks. The improved effect of the I-HA, compared to I-CS, persisted until 26 weeks. That is, I-HA provides improved duration of benefit compared to I-CS, which was also demonstrated by Rodriquez-Merchan [52]. Smith et al. compared the WOMAC score of patients treated with I-HA to those treated with a combined I-HA/CS and found that those treated with the combination injection had improved pain scores at up to 52 weeks, but no change in total WOMAC scores [53]. Repeated courses of I-HA appear to be an effective and safe treatment of K-OA. A 2018 systemic review examined patients treated with recurrent courses of I-HA for up to 25 months and found that the most common side effect of repeated I-HA was joint swelling and arthralgia [54]. HA preparations of various molecular weights are available in North America, and most meta-analysis and systemic reviews lump the various HA preparations together when analyzing the data. Johan et al. sifted through the discordant conclusions and found that the use of high molecular weight I-HA preparations led to clinically important reductions in pain [48]. Further large trials assessing response in K-OA to I-HA across various HA preparations are needed. Until then, the data remain heterogeneous, and the current guidelines reflect the methodological challenges of available primary evidence.

## 6. Platelet-Rich Plasma

I-PRP is a product of regenerative medicine that is gaining much clinical interest. PRP is autologous plasma that has been prepared to contain a higher concentration of platelets than *in vivo* plasma. While there is no standardized concentration of platelets in PRP, it is generally accepted that it should contain somewhere between 2 and 8 times the concentration of platelets as autologous serum [29, 35]. When platelets are activated, they rapidly release numerous growth factors, such as TGF-*β* and IGF-1, from their *α*-granules [55]. Together with coagulation factors, cytokines, and other platelet proteins, these growth factors are thought to act on chondrocytes and enhance the chondrocyte cartilaginous matrix as well as diminish the inflammatory effects of certain cytokines involved in the process of OA [29]. Certain growth factors continue to increase at various time points after I-PRP, insinuating that PRP stimulates endogenous pathways rather than simply delivering a one-time hit of growth factors [56]. PRP is obtained by centrifuging autologous patient blood, using either a one or a two-spin approach. This will generate either leukocyte-poor (LP) or leukocyte-rich (LR) PRP, respectively [29, 35]. No clear benefit has been established between using either LP-PRP or LR-PRP, but adverse events seem to be more common with LR-PRP [35, 57]. This might be secondary to the richer concentration of leukocytes present in LR-PRP and the stronger induced pro-inflammatory response [35]. Nonetheless, adverse events after all I-PRP interventions were minor, including injection site pain and joint stiffness [35]. The lack of a standardized protocol in the literature is a matter of much consternation for reviewers and likely contributes to variable data and outcomes.

I-PRP begins to provide pain control about 2 months after injection and may last as long as 12 months [35, 58–60]. There is some suggestion based on a guinea pig model that multiple, weekly I-PRP injections provide more sustained benefit than a single I-PRP injection [61]. There is a paucity of high quality date comparing I-PRP use in K-OA to I-CS as well as to NSAIDS, which remain the only treatment modality recommended by the AAOS in 2013 [29]. A 2019 double-blind, level I randomized control study conducted by Di Martino et al. in Bologna over a five-year period demonstrated that both I-HA and I-PRP had both similar efficacy and duration of effect [62]. When the primary outcome was the WOMAC score, a level I randomized control trial by Cole et al. similarly found that there was no difference between I-HA and I-PRP. However, when other patient outcome measures were examined, they found PRP provided a significant improvement in outcomes at 24 and 52 weeks. They hypothesized that this was because the WOMAC focused on low activity levels and could not discern clinically significant differences in their younger cohort (mean age for I-HA was 56.8, and mean age for I-PRP was 55.9) [63]. A systemic review by Laver et al. in 2017 also demonstrated a clear benefit for I-PRP vs. I-HA for K-OA in 9 out of 11 of the studies reviewed. Furthermore, they found a trend for better results for I-PRP in younger patients with early K-OA [64]. This has been supported by other meta-analyses and systemic reviews and studies [58, 59, 65]. Compared to I-HA, Meheux et al. found that patients receiving I-PRP had significantly decreased WOMAC scores at both 3 and 6 months (28.5 and 43.4, respectively, *P* = 0.0008) as well as at 6 and 12 months (22.8 and 38.1, respectively, *P* = 0.062) [60]. This is in contrast to a therapeutic study by Duymus et al. who found that I-HA and I-PRP had similar improvements in WOMAC score at both 1 and 6 months after injection; however, by 12 months after injection, I-PRP was both statistically and clinically superior to I-HA [66]. In summary, I-PRP is a promising new product of regenerative medicine that may be superior to our current therapies but, unfortunately, lacks robust evidence to support its use in clinical practice. This therapy is not covered by insurance plans and, as currently prepared, likely will not ever be as it is created by centrifugation of the patient's own blood through a multitude of different ways to create a nonstandardized final product.

## 7. Prolotherapy

Often included in the regenerative therapies, prolotherapy is a relatively uncommon treatment for treating K-OA with very little scientific evidence. However, as a relatively simple and inexpensive treatment modality with a high safety profile, prolotherapy is something that could easily be performed in the primary care setting and is thus worth our consideration [67–70]. A hypertonic irritating solution is used to treat the affected knee and can be injected both peri- and intra-articular. Often this is dextrose, and it is injected both intra- and peri-articular [68]. A small study published in 2017 showed no significant changes in WOMAC and VAS scores between patients treated with either intra- or peri-articular prolotherapy [67]. There are some variabilities in the method of peri-articular administration of the dextrose, but the two key techniques are either Lyftogt's technique or Hackett's technique [69]. In Lyftogt's technique, dextrose is injected into subcutaneous tissues. In contrast, Hackett's technique involves injecting dextrose into the fibro-osseous junction of ligaments or tendons. A small benefit has been observed when a combination of both techniques is performed [69, 71].

The exact mechanism of prolotherapy is not well understood, but it is thought to induce a pro-inflammatory response that results in the release of growth factors and cytokines, ultimately resulting in a regenerative process within the affected joint [69, 72]. Injection of the hyperosmolar dextrose solution might also hyperpolarize nociceptive pain fibers by forcing open potassium channels, resulting in reduced pain perception [69]. Animal studies have shown that peri-articular dextrose injection may also promote recovery by leading to vascular and fibroblast proliferation and cartilage thickening [68].

The efficacy of prolotherapy is also unclear; multiple systemic reviews and meta-analysis have attempted to examine the same and have been marred by the low quality and heterogeneity of the available studies. Sit et al. examined three randomized control trials and one quasi-randomized trial to conclude that prolotherapy led to reduced WOMAC scores in patients with K-OA compared to exercise [68]. Unfortunately, all confidence intervals crossed one, calling into question the robustness of this claim. A systemic review in 2017 examined ten studies for the efficacy of prolotherapy in K-OA and concluded that while all studies demonstrated positive outcomes and high patient satisfaction after prolotherapy, meta-analysis was not possible because of high data heterogeneity [69]. Krsticevic et al. also came to similar conclusions [72]. In sum, prolotherapy likely provides at least some benefit, although the quality of available data makes this statement hard to prove and it certainly does not cause harm.

## 8. Stem Cell Therapy

Over the last decade, stem cells have emerged as a therapy for treatment of pain and regeneration of cartilage. There have been attempts to use stem cells to reverse the damages of K-OA. As the pathophysiology of K-OA is considered both degenerative and inflammatory, stem cell therapies are hoped to promote tissue regeneration by enhancing local growth factors and promoting an anti-inflammatory immune response [73, 74]. Mesenchymal stem cells (MSC) are easily harvested from adipose tissue, bone marrow, and even placental tissue, and therefore most research has focused on this cell progenitor [75–78]. MSC produce growth factors such as TGF, VEGF, and FGF that allow for the induction of various cell types and tissue repair. IGF, IL-6, as well as the abovementioned growth factors also play a role in preventing cell apoptosis [74, 79, 80]. Through a complex process, MSCs exert their effect by promoting an anti-inflammatory and immunosuppressive role [81, 82]. They also have the potential to differentiate into chondrocytes and therefore have a theoretical advantage in joint repair [77]. It is speculated that MSCs can attract cells to degenerative sites and lead to a proliferation of a functional cartilage. MSC may also repopulate cell progenitors to reduce inflammation or further degradation. Yet, the exact mechanism of action remains unaddressed in the clinical literature.

Review of the literature prior to 2015 reported mostly beneficial effects of stem cell therapy in K-OA, but these reviews were determined to be poorly designed systematic trials, and included animal models [75, 77, 83–86]. In 2016, Chala et al. conducted a systematic review and included 6 studies (and 300 knees)—three studies on treatment of OA and three studies on treatment of focal cartilage defects. Despite positive results in each trial in reducing pain and improving function, they varied with respect to cell sources, cell characterization, additional adjuvant therapies (such as I-PRP and I-HA), and assessment tools. The placebo effect of stem cell injections was not considered [87]. A recent systematic review by Pas et al. evaluated five randomized controlled trials and one non-randomized controlled trial and found the data to be so heterogeneous, and at such high risk of bias, a meta-analysis was not even possible [75].

Studies have proven the safety and tolerability of this therapy with no reported infections or adverse events [88–90]. However, questions and cautions surround every other aspect of this therapy. Little is known regarding the specific indications for stem cell therapy in K-OA, optimal cell sources, or preparation and delivery methods that allow for a consistent cell culture. Procuring stem cells through allogeneic placental tissue raises ethical questions, and autologous procurement from bone or fat may require an anesthetic due to its invasive nature. MSCs that are taken from patients with end-stage OA lack the differentiation and proliferation potential as those taken from people without OA and may not be as effective [91]. Long-term studies are needed to assess both effect and safety of stem cell therapies in K-OA.

## 9. Genicular Nerve Blocks

Neuronal innervation of the knee is multipart, involving the articular branches of the femoral, tibial, saphenous, obturator, and common peroneal nerves [92–94]. The culmination of these articular branches is referred to as the genicular nerves. Using precise anatomic locations, at the superior lateral, superior medial, and inferior medial borders at the periosteal areas of the femur and tibia, pain physicians can alter these nerves using radiofrequency ablation (RF).

RF ablation of the genicular nerves is a minimally invasive technique to treat K-OA. The procedure creates a high frequency current created by an electrode at the needle tip. A current passes from the electrode through a grounding pad placed on the body. The electromagnetic field created at the distal end of the needle produces heat that causes protein denaturation and/or cell necrosis depending on the temperature, duration, and vicinity of the active needle tip to the nerve [95, 96]. Pain relief is achieved by inhibiting the genicular nerve fibers that innervate the knee joint. There are numerous approaches from conventional RF ablation (high-temperature nerve ablation), pulsed RF (low-temperature electrical stimulation), and the inaptly-named cooled RF ablation (high-temperature nerve ablation) to alter neuronal structures and chemistry for an extended duration [92].

Despite this therapy being available for decades, there is limited evidence in the literature describing its benefit. A retrospective small trial reviewing pulsed RF for K-OA in 29 patients found a significant improvement in WOMAC scores after 12 weeks without adverse events [97]. Similar results with RF ablation in 26 patients found average pain relief improvement by 67% based on the Visual Analog Scale at 3 months, and for those who had a good effect with the procedure continued to described pain relief at 6 months [95]. Neither of these small trials had a control group, and a small sample size makes it difficult to implement in clinical practice. Larger trials such as a systematic review by Gupta et al. in 2017 compared conventional, pulsed, and cooled radiofrequency ablation. Despite evidence for RF ablation effect for up to one year, the authors determined that the small study sizes, inconsistent patient assessment measures, and procedure methodology made it impossible to support the superiority of any specific RF procedure modality [92]. In 2018, Davis et al. conducted a prospective randomized crossover trial comparing effectiveness of cooled RF versus an I-CS injection in 151 patients at 1, 3, and 6 months [98]. With similar effect on pain relief and function at 1 month, the study found statistically significant differences at 3 and 6 months. At 6 months, 74.1% of patients in the cooled RF ablation group reported a >50% reduction in pain based on a numerical-rating scale, compared to only 16.2% in the corticosteroid group (*P* < 0.0001). Knee function and an overall perceived benefit were greater in the cooled RF group vs. corticosteroid group. RF ablation can be considered a safe, nonopioid, and non-CS option for K-OA with lasting benefit, but evidence to support its superiority over other therapies is lacking.

## 10. Discussion

In a Canadian healthcare era, where clinical practice guidelines direct treatments based on cost and efficacy, much of the injection-based therapy for K-OA still remains controversial, and higher-level evidence will be necessary to tease out superior therapies in the future. The current literature on injection therapies contains numerous small studies, with mostly positive results on reducing pain and improving function. The included systematic reviews attempted to assess many of the injection therapies, and all concluded that the studies were heterogeneous and of low quality. Between studies, each therapy was used in a variety of doses, strengths, preparations, and at varying intervals making statistical significance difficult to achieve. Many studies did not use a control population or account for the concurrent use of oral anti-inflammatories, while often using poorly defined protocols for the study treatment.

It is difficult to account for a placebo response and use of a true control therapy in pain medicine trials. An increased magnitude of effect from a placebo response diminishes the effect size of the treatment arm. Intra-articular normal saline, a common control and placebo, has been shown to demonstrate biological activity and objective improvement in WOMAC scores [99]. This further confounds the data of effectiveness for intra-articular therapies as many studies reviewed included saline as a control group, whereas other studies did not include any control group.

Despite the limitations of the data, I-PRP has shown promising results in both duration and effect in patients with mild K-OA, when compared to I-HA and other therapies [64]. Currently, in Canada, this therapy is not covered, and out-of-pocket expenses make it difficult to offer to the average patient. In addition, there are a variety of companies selling devices for I-PRP production, and there is no evidence to determine device superiority. If I-PRP continues to show superiority compared to other therapies, the next step to creating an affordable and reliable treatment will be to standardize the manufacturing process of relevant growth factors.

The use of I-CS for the treatment of K-OA should be reconsidered. Despite it being one of the most common insurance-covered injection therapies in Canada, it has limited evidence of efficacy. Yet interestingly, many of the major societies continue to approve its use based on limited evidence (Table 1). The Cochrane review by Juni et al. places this therapy into controversy [37]. Even though I-CS is cheap to administer, the risks of corticosteroids may outweigh the benefits in K-OA. However, until alternate treatments are approved for use by Health Canada and covered by medical insurance, it will likely remain a first-line intra-articular therapy used by physicians.

Better injectable treatments are needed for K-OA, and new therapies emerge yearly in the United States. Recently, the use of cryoanalgesia to create a cooled area around the nerves that innervate the knee has shown promise in treating knee-OA [100]. Alternately, peripheral neuromodulation devices that use electricity to stimulate nerves and decrease sensation in painful joints could theoretically be used to treat knee-OA and other nonoperative painful joints [101]. The use of multiomics, primarily proteomics, and metabolomics is a promising new approach in K-OA. Already, several components of the classical complement cascade as well as pro-inflammatory cytokines (e.g., IL-6, IL-8, and IL-18) have been identified in the synovial fluid of K-OA [102]. Identification of these relevant biomarkers will be helpful in early diagnosis of K-OA and in creating novel K-OA treatments. Lastly, Resiniferatoxin, a new compound for pain relief, is currently being studied in patients with moderate-to-severe K-OA. [103] While promising, these new treatments will not have the evidence to support their use for many years.

The treatments for K-OA reviewed above are relative to TKA, inexpensive, as well as simple to perform with excellent safety profiles. Despite this, statistical significance of efficacy has proven difficult to attain secondary to high interstudy heterogeneity. Future research will be needed to corroborate the promising clinical results shown for many of the injectable therapies, such as I-PRP.

## Figures and Tables

**Table 1:** Pharmacological and procedural recommendations for treatment of knee osteoarthritis.

Society	Recommended	Inconclusive	Not recommended
Arthritis Alliance of Canada/College of Family Physicians of Canada	(i) Topical NSAIDs (ii) Acetaminophen (iii) Oral NSAIDs (in patients without contraindications) (iv) SNRI (i.e., duloxetine) (v) Opioids (tramadol is preferred as nontramadol opioids having a higher incident of side effects with small effects on pain) (vi) I-CS	(i) Herbal medications (ii) I-HA (iii) I-PRP (iv) Stem cells	(i) Capsaicin (ii) Chondritin (iii) Neuropathic pain modulators (iv) Glucosamine
American Academy of Orthopedic Surgeons	(i) Topical NSAIDs (ii) Oral NSAIDs (iii) Tramadol	(i) Acetaminophen (ii) Nontramadol opioids (iii) I-CS (iv) I-PRP	(i) Chondritin (ii) Glucosamine (iii) I-HA
Osteoarthritis Research Society International	(i) Topical NSAIDs (ii) Capsaicin (iii) Acetaminophen (in patients without comorbidities) (iv) Oral NSAIDs (in patients without comorbidities) (v) SNRI (i.e., duloxetine in patients without comorbidities) (vi) I-CS	(i) Chondritin (for symptom relief) (ii) Glucosamine (for symptom relief) (iii) Opioids (iv) I-HA	(i) Chondritin (for disease modification) (ii) Glucosamine (for disease modification)
American College of Rheumatology	(i) Topical NSAIDs (ii) Acetaminophen (iii) Oral NSAIDs (iv) Tramadol (v) I-CS	(i) SNRIs (i.e., duloxetine) (ii) Nontramadol opioids (iii) I-HA	(i) Topical capsaicin (ii) Chondritin (iii) Glucosamine

**Table 2:** Overview of the Western Ontario and McMaster Universities Osteoarthritis Index scoring system (WOMAC). Items in each category are assigned a numerical score from zero to four, with higher scores indicating greater disability.

Category	Description of items in each category
Pain	5 items: standing upright, using stairs, sitting or lying, during walking, and in bed
Stiffness	2 items: after waking and later in the day
Physical function	17 items: light domestic duties, heavy domestic duties, getting on/off toilet, lying in bed, rising from bed, getting in/out of bath, getting in/out of car, putting on socks, taking off socks, walking on a flat surface, bending to floor, descending stairs, ascending stairs, sitting, rising from sitting, standing, shopping
